# Innovative Approach for Controlling Black Rot of Persimmon Fruits by Means of Nanobiotechnology from Nanochitosan and Rosmarinic Acid-Mediated Selenium Nanoparticles

**DOI:** 10.3390/polym14102116

**Published:** 2022-05-23

**Authors:** Mohamed F. Salem, Ahmed A. Tayel, Fahad Mohammed Alzuaibr, Ramadan A. Bakr

**Affiliations:** 1 Department of Environmental Biotechnology, Genetic Engineering and Biotechnology Research Institute, University of Sadat City, El-Sadat City 32897, Egypt; mohamed.salem@gebri.usc.edu.eg; 2 Department of Fish Processing and Biotechnology, Faculty of Aquatic and Fisheries Sciences, Kafrelsheikh University, Kafrelsheikh City 33516, Egypt; 3 Department of Biology, Faculty of Science, University of Tabuk, Tabuk 71491, Saudi Arabia; falzuaiber@ut.edu.sa; 4 Plant Pathology Branch, Department of Agricultural Botany, Faculty of Agriculture, University of Menoufia, Shibin El-Kom 32514, Egypt; ramadanbaker82@agr.menofia.edu.eg

**Keywords:** *Alternaria* black rot, antifungal, edible coatings, *Diospyros kaki*, firmness, nanomaterials, nanopolymers, postharvest

## Abstract

The protection of persimmon fruits (*Diospyros kaki* L.) from postharvest fungal infestation with *Alternaria alternata* (*A. alternate*; black rot) is a major agricultural and economic demand worldwide. Edible coatings (ECs) based on biopolymers and phytocompounds were proposed to maintain fruit quality, especially with nanomaterials’ applications. Chitosan nanoparticles (NCt), rosmarinic acid bio-mediated selenium nanoparticles (RA/SeNPs) and their composites were produced, characterized and evaluated as ECs for managing persimmon black rot. The constructed NCt, RA/SeNPs and NCt/RA/SeNPs composite had diminished particles’ size diameters. The ECs solution of 1% NCt and NCt/RA/SeNPs composite led to a significant reduction of *A. alternata* radial growth in vitro, with 77.4 and 97.2%, respectively. The most powerful ECs formula contained 10 mg/mL from NCt/RA/SeNPs composite, which significantly reduced fungal growth than imazalil fungicide. The coating of persimmon with nanoparticles-based ECs resulted in a significant reduction of black rot disease severity and incidence in artificially infected fruits; the treatment with 1% of NCt/RA/SeNPs could completely (100%) hinder disease incidence and severity in coated fruits, whereas imazalil reduced them by 88.6 and 73.4%, respectively. The firmness of fruits is greatly augmented after ECs treatments, particularly with formulated coatings with 1% NCt/RA/SeNPs composite, which maintain fruits firmness by 85.7%. The produced ECs in the current study, based on NCt/RA/SeNPs composite, are greatly recommended as innovatively constructed human-friendly matrix to suppress the postharvest destructive fungi (*A. alternata*) and maintain the shelf-life and quality of persimmon fruits.

## 1. Introduction

Persimmon fruits “*Diospyros kaki* Thunb.” are the nutritionally, wide-distributed species in Asia, southern America and Mediterranean regions [1]. These precious fruits can provide many nutritional, bioactive and health-promoting agents, e.g., vitamins, minerals, dietary fiber, polyphenols, carotenoids, flavonoids, terpenoids and steroids, which have great potential for human health [2]. The persimmon global production was intensified five times during the last two decades, with a great increase in their cultivation area [3]. The persimmons have climacteric manners, comprising rapid ethylene production and increased respiration rate, which resulted in limited marketability period and fast fruits’ spoilage [2].

The storage of persimmons at <10 °C can commonly compromise their quality, e.g., peels translucence and mottling, flesh darkening and gelling, flavor and juice loss and production of off-odors [1]. The firmness loss in persimmon is principally attributed to polymerization and solubilization of pectin contents, cellulose decomposition and cellular oxidation/peroxidation processes during ripening; these factors are mainly enzymatically dependent [4].

Of the diverse factors for harvested fruits and vegetable spoilage, a fungal attack is a leading factor that causes the majority of crop losses [5]; these destructive fungal pathogens can sporulate, resist most postharvest treatments and infect the fruits at any stage from farming to marketing. *Alternaria alternata* is one the most aggressive fungal species that can attack plentiful agricultural produces, in diverse climatic conditions [6]. *A. alternata* is also associated with food poisoning/toxication, as it can secrete multiple mycotoxin types (e.g., alternariol, alternariol monomethyl ether, L-tenuazonic acid, attenuate and altertoxins) during its growth on many crops [7].

Edible coatings (ECs) are the emerged promising tool for foodstuff preservation; they were defined as “thin layers of materials, which cover foodstuff surface and could be eaten and deemed as a part of the entire food product” [8]. The ECs were widely reviewed to extend the shelf-lives and storage of fresh cuts (fruits and vegetables) [8,9,10,11]. Many polymeric materials (e.g., chitosan, alginate, cellulose, agar, gum Arabic, etc., were employed for forming ECs to provide barriers toward moisture, microbes, O_2_ and CO_2_; thus can manage the physiological functions of crops [9]. The ECs, in their liquid forms that contain edible materials, can be amended with further bioactive compounds and nanoparticles to augment their preservative actions [10]. In addition to their parrying actions, ECs can provide many supplementary sensory enhancers, nutrients, antimicrobial agents and nutraceuticals, while being consumed on food coverage [11].

Plentiful types of bioactive compounds were successfully incorporated into the ECs matrix to augment their antimicrobial and antioxidant potentialities. This could reduce the texture and enzymatic breakage during storage [10]. The constructions of ECs materials that contain metal nanoparticles (NPs) have attained promising success for providing extra antifungal, antioxidant and tissue protection potentialities [10,12,13]. The ECs materials are required to contain only eco-friendly and GRAS “Generally Recognized as Safe” components that have minimal biotoxicity to humans, and further could provide many nutritional and functional elements to enrich the human body [8,10].

Chitosan (Cht), the biopolymer that is derived from chitin deacetylation, is reported to possess numerous bioactivities and potentialities for applications as a human-friendly compound [14]. Cht has numerous extraordinary attributes, e.g., its cost-effectiveness, biocompatibility, biosafety, biodegradability and molecules-carrying activities, which enabled its applications in environmental, biomedical, therapeutic, and nutritional aspects [14,15]. The Cht antimicrobial potentialities were demonstrated toward diverse microbial genera/species from bacteria, yeast or fungi [15]. The NPs formation from Cht (e.g., NCt) provided extra functionalities and bioactivities for this amazing biopolymer; the NCt was effectually employed as a potent antimicrobial, anticancer, drugs’ carrier, toxicant adsorbent, antioxidant, metals’ binder, anti-inflammatory, and nutraceuticals carrier [16,17,18,19,20].

Phytochemicals are the plant-derived molecules/composites that possess numerous beneficial bioactivities and impacts on human health, e.g., their antimicrobial, antioxidant, anticancer, anti-inflammatory and immune-support [21]. The polyphenols are from the most precious phytochemicals that could benefit human nutrition and health [22]. Rosmarinic acid (RA) is the ubiquitous phenolic that belongs to hydroxycinnamic acid family and is found in >30 plant families; RA has many proven pharmacological and biological potentialities [23]. The elevated RA antioxidant activity was attributed to its constituents from catechol groups, but RA has poor bioavailability because of its instability, inadequate permeability, and water insolubility, which obstruct its practical employment [24]. However, the loading of RA onto functional nanomaterials (e.g., NCt) can solve these challenges and provide extra functionality to the composite [25,26].

Of the highly important elements for biological functions, selenium (Se) plays vital roles as a prooxidative, co-enzymes and antioxidative agent; Se is required at 40–300 μg/day range to provide man with essential requirements [27]. The key vital functions of Se include enzymes’ activation, free radical protection, cellular metabolism, thyroid metabolism, human fertility, and further energetic functions. The Se nanoforms (SeNPs) could be generated via various protocols (chemically, physically and biologically); the SeNPs possess surplus bioactivities over bulk metal, e.g., lower toxicity and higher bioactivities [28,29]. Therefore, particularly bio-synthesized SeNPs were employed in numerous bio-applications, e.g., in therapeutic, biomedical and nutritional fields, containing the SeNPs practices in antimicrobial, anticancerous, anti-infective, antioxidants, cytokine inducers, enzyme inhibitors, and immune-modulatory formulations [29,30].

No obtainable former studies could be attained about the biosynthesizing of SeNPs with RA and their conjugation with NCt to formulate ECs to protect harvested fruits and agricultural crops. Thus, the targeted nanocomposites here could be proposed as an innovative matrix for inhibiting fungal destruction and sustaining persimmon quality.

Accordingly, the synthesis of different NPs (NCt, RA-mediated SeNPs, and their nanocomposites) was intended here, to formulate potential antifungal and ECs composites, to prohibit *A. alternata* growth and maintain the shelf-life and quality of persimmon fruits. These could be greatly noteworthy worldwide to control this destructive fungal disease of persimmon via nanobiotechnology.

## 2. Materials and Methods

### 2.1. Materials

Chitosan (Cht) is extracted from *Fenneropenaeus indicus* (white prawn) shells and has a molecular weight of ~38 kDa, and a deacetylation percentage of 89.4% [18] was experimented with in this study. The entire used chemicals/reagents in experimentation had certified grades, e.g., rosmarinic acid, sodium selenite (Na_2_SeO_3_–5H_2_O), acetic acid, ethanol, ascorbic acid, tripolyphosphate (TPP), imazalil, Tween 80 and microbiological media; were acquired from Sigma-Aldrich Co. (St. Louis, MO, USA). Milli-Q water (MQ, Millipore, Milford, MA, USA) was used for conducting experiments.

### 2.2. Nanocomposites Fabrication

The synthesis of targeted nanomaterials/nanocomposites was conducted using eco-friendly materials to warrant their biosafety and innocence for human usage.

The fabrication of NCt that encapsulated RA-mediated SeNPs followed the ionic-gelation method, as adopted from Almutairi et al. [16]. Stock solutions were prepared from Cht (1.0% concentration, *w*/*v* in 15 mL/L acetic acid aqueous solution), RA (0.1%, *v*/*v*, in absolute ethanol), sodium selenite (1 mM in MQ), TPP (0.1%, *w*/*v*, in MQ) and ascorbic acid (1.0, *w*/*v*, in MQ). Each solution was sonicated in an ultrasonic bath (Grant instruments, Cambridgeshire, UK) for complete dissolution. For NCt/RA nanocomposite preparation, (1) 50 mL of Cht and RA solutions were intermingled with the addition of 1.2 mL Tween 80 and vigorously stirred (750× *g*) for 90 min, then (2) 100 mL of TPP solution was slowly dropped into the mixed solutions (at 0.4 mL/min rate) while stirring. (3) The sonication was performed after complete TPP dropping for 10 min, then (4) the formed nanoparticles were harvested via centrifugation (at 11,200× *g*, 2–16 KL, SIGMA, Osterode am Harz, Germany). The NPs pellet was washed with MQ and ethanol, recentrifuged and lyophilized. For synthesizing NCt/RA/SeNPs nanocomposite, 25 mL of sodium selenite solution and 10 mL of ascorbic acid solution were intermingled with the solutions in step (1) and the rest of the steps were continued as above.

### 2.3. Nanoparticles Characterization

The structural and biochemical attributes of synthesized nanoparticles/nanocomposites (e.g., NCt, NCt/RA and SeNPs) were evaluated.

#### 2.3.1. FTIR Analysis

The infrared analysis of produced molecules could validate their biochemical structures and interactions via detecting their biochemical bonding. The “Fourier transform infrared spectroscopy” spectroscopy (FTIR; FTS 45, Biorad, Munich, Germany) was employed for assessing the infrared spectra of NCt, RA and NCt/RA/SeNPs. The transmittance of samples was appraised within 400–4000 cm^−1^ wavenumber, after amalgamating each sample with 1% KBr [24]. The analysis was performed at 22 °C and at a resolution of 4 cm^−1^, each dried sample was finely ground and mixed well with anhydrous KBr, then amalgamated samples were positioned for FTIR analysis.

#### 2.3.2. Zeta Potential and Particles’ Size (Ps) Distribution

The Zetasizer “Malvern Nano ZS instrument, Southborough, MA” was used for appraising the surface charges (zeta potential) and distribution of nanoparticles/nanocomposites Ps, using the Dynamic Light Scattering (DLS) technique, for synthesized NCt, NCt/RA, NCt/RA/SeNPs and SeNPs [26]. The dissolved samples in DW were sonicated for 35 min prior to investigation, the hydrodynamic diameters and the zeta potentials of screened particles were measured in backscatter configuration (θ = 173°) at λ = 633 nm laser wavelength. The attained data were analyzed using Zetasizer software V. 7.03.

#### 2.3.3. Scanning Electron Microscopy (SEM) Imaging

The SEM imaging “JEOL JSM- IT100, Tokyo, Japan” was used for surface and structural morphology screening for the SeNPs and NCt/RA nanocomposite. The used conditions for SEM imaging included the accelerator voltages of 10–15 kV and magnifications of 10,000–30,000×. However, the energy of the accelerator beam was fixed to provide the maximum potential resolution of detected samples.

### 2.4. Effect of Nanomaterials on Growth of Alternaria Alternata In Vitro

*Alternaria alternata* (Fries) Keissler (ATCC-44498) was employed to assess the antifungal potentiality of synthesized nanoparticles/nanocomposites. The fungal culture was propagated and screened using Potato Dextrose Broth and Agar (PDB and PDA, respectively), aerobically at 25 °C. The nanomaterials (NCt and NCt/RA/SeNPs), at concentrations of 0.5 and 1.0% (*w*/*v*), were appraised for their competence to suppress *A. alternata* growth in vitro.

The nanomaterials were amended to PDA and 5 mm mycelial discs were cut from the periphery of seven-day-old cultures of *A. alternata* and centrally positioned on surfaces of control and NPs-amended plates. The plates were conditioned for 7 days at 25 °C, and the developing colonies’ diameters were measured. The reductions (%) of colony radial growth percentage were calculated via the next formula [26]:

Reduction % = (D_c_ − D_t_)/D_t_ × 100 (1)

where D_c_ and D_t_ are the mean fungal colonies diameters, in the control and treated sets, respectively.

### 2.5. Persimmon Treatment with Nanoparticles-Based Edible Coating

For assessing the efficacy of formulated ECs to protect persimmon fruits from black rots and maintaining their quality, organically farmed *Diospyros kaki* L. (Persimmon) fruits were collected at the harvest stage from a certified farm in Kafr El Dawar, Beheira Governorate, Egypt. The fruits were selected with uniform size, shape and color; they were free from any infestation or damage signs. The fruits were firstly washed and disinfected with 70% ethanol for 70 s. Each fruit was wounded via puncturing with a sterilized metal rod (0.5 mm diameter × 2 mm depth) around the equator [26].

The artificial infections were performed via preparation *A. alternata* spores’ suspension (~4 × 10^5^ spores/mL) after scrapping 7-day-old grown mycelia on PDA and washing them with MQ water then adjusting the spores number with hemocytometer counting.

The fruits inoculation involved their dipping in spores suspension for 15 s, draining, and air-drying for 25 min.

The ECs preparation involved the addition of NCt or NCt/RA/SeNPs (at 0.5 and 1.0% concentrations) in MQ water and sonication for complete dissolving, then the fruits were immersed in ECs for 2 min and left to dry. The fruits sterilized water and the imazalil-containing solution (0.5%) were the controls for the experiments [26].

Each group consisted of 20 fruits and 3 replicates were prepared from such groups. The treatments were packed inside penetrated carton boxes and stored at 15 ± 1 °C with 92–95% relative humidity for 30 days.

### 2.6. Determination of Black Rot Disease Development Incidence and Severity

The incidence and severity of *A. alternata* black rot disease in artificially infected persimmon fruits were assessed after 21 days of storage to judge the effectiveness of ECs in preventing disease progression. The incidence of disease involved the number of fruits with infection signs and calculated as percentages from the number in the control (water-dipped) group. The disease severity involved the determination of mean lesions diameters (mm) around wounds in each group, and their results were calculated as reduction percentages of severity, with respect to water-dipped fruits [31].

### 2.7. Statistical Analysis

Triplicated experiments were performed; statistical software (SPSS V 11.5, Chicago, IL, USA) was used for calculating means ± SD (standard deviation) using a *t*-test and one-way ANOVA; the differences’ significances were handled at *p* ≤ 0.05.

## 3. Results and Discussion

### 3.1. Biomaterials FTIR Analysis

**Figure 1:**
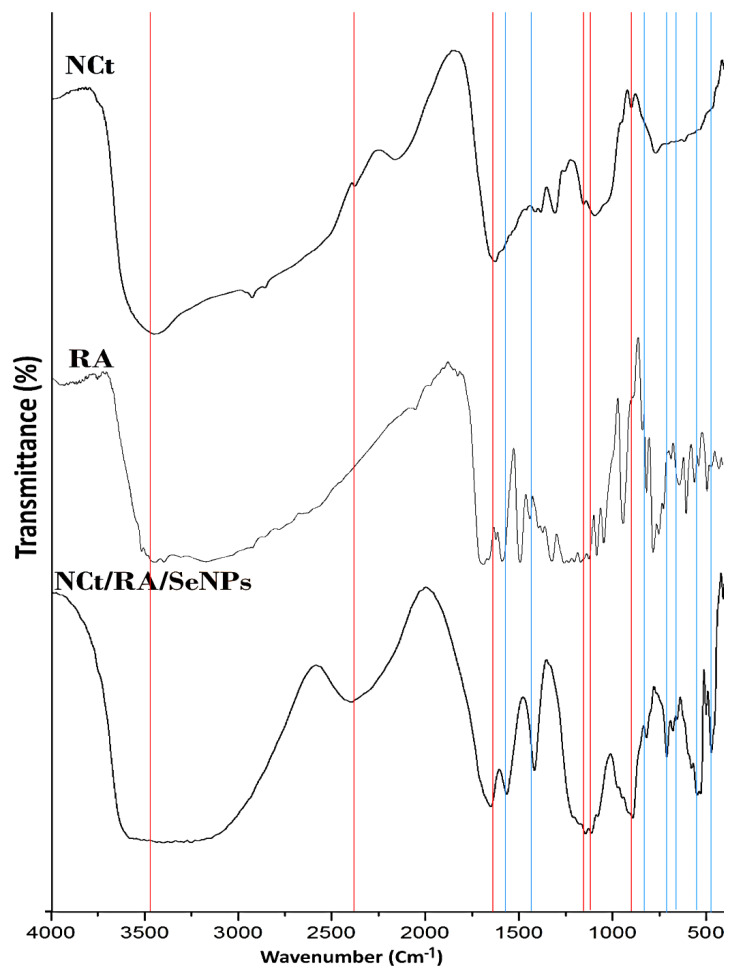
FTIR spectra of screened molecules including chitosan nanoparticles (NCt), rosmarinic acid (RA) and their composites with SeNPs*; The vertical red lines indicate the derived peaks from NCt and the blue lines indicate the derived peaks from RA.

The extracted and fabricated nanomaterials/nanocomposites were characterized for their biochemical and structural attributes, other than their bioactivity for preserving persimmon fruits. The biochemical physiognomies of screened compounds (NCt, RA, and NCt/Ra/SeNPs) were spectroscopically investigated via FTIR (Figure 1), to elucidate their key bonds and interactions. For NCt (Figure 1—NCt), the infrared analysis designated the existence of most characteristic bonding in native Cht, which were demonstrated formerly [28,32]. Moreover, the key distinctive bonds/groups in the NCt spectrum were positioned at 3422 cm^−1^ (overlapped N–H and O–H extensional vibrations, 1414 cm^−1^ (C–H vibrated stretching), 2911 cm^−1^ (C–H vibrated stretching), 1086 cm^−1^ (C–O–C), and at 901 cm^−1^ (pyranose ring). Furthermore, the designated peak for chitosan-TPP binding and formation of P=O linkage was identified at 1252 cm^−1^, authorizing NCt transformation [28,33]. From the additional NCt designated peaks were those detected at 1683 cm^−1^ (stretching C=O of amide I), 1610 cm^−1^ (N–H vibrated stretching of amide II), 1107 cm^−1^ (C_3_–OH vibrated stretching) and 1036 cm^−1^ (C_6_–OH vibrated stretching) [34,35]. The above-detected peaks in NCt could confirm the successfulness of chitosan extraction and its effectual transformation into nano-form.

The RA spectrum (Figure 1—RA) displayed the typical vibrational bands, including the key bands located within 1700 and 600 cm^−1^ [36]. The distinctive bands at 1603, 1596, and 1447 cm^−1^ are attributed to the existence of aromatic rings’ stretching in RA molecules [25]. In addition, the phenolic groups in RA were indicated through the 1364 and 1178 cm^−1^ bands, which appointed the O–H and C–O stretches, respectively [36]. Additionally, the RA infrared spectrum displayed other designative bands, e.g., at 3454 and 3398 cm^−1^ (stretching –OH in phenolic groups); 3191 cm^−1^ (C–H stretching); 1726 and 1704 cm^−1^ (C=O stretching); 1634 cm^−1^ (C=C stretching); 1603 (aromatic C–C stretching); 859 and 687 cm^−1^ (aromatic C–H) [37]. The detected bonds/groups in the RA spectrum are the main responsible for its biochemical activities, e.g., antioxidant and antimicrobial activities [23]. In addition, these groups can interact with further biomolecules’ groups (e.g., in biopolymers) to form stable conjugates between RA and them [25,26].

Numerous NCt and RA distinctive peaks were appeared in NCt/RA/SeNPs composite spectrum, as designated with vertical red lines for the derived peaks from NCt and the blue lines for the derived peaks from RA (Figure 1-NCt/RA/SeNPs), which strongly confirmed the existence of both molecules in the nanocomposite. Many peaks from both composited molecules, especially in the range of 760–1390 cm^−1^, either disappeared or became less intense in the composite spectrum, which indicates the occupation of their active bonds after interactions between NCt and RA/SeNPs particles [24]. Accordingly, the FTIR spectrum of NCt/RA/SeNPs could validate the various interactions (electrostatically and biochemically), especially between NCt amino groups and RA carbonyl groups [25,26].

### 3.2. Nanomaterials Structural Analysis

**Figure 2:**
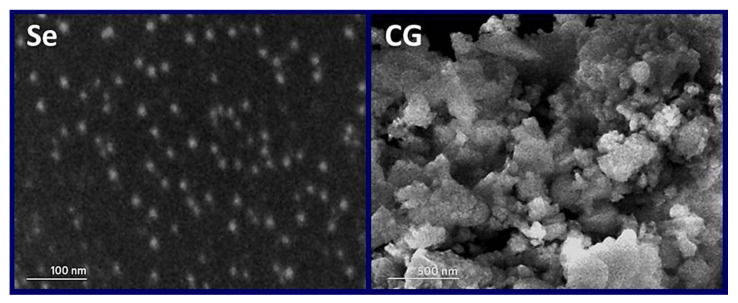
Scanning features of synthesized nanoparticles, including plain synthesized selenium nanoparticles (**Se**) and encapsulated rosmarinic acid/SeNPs into chitosan nanoparticles (**CG**).

The structural attributes of generated nanoparticles/nanocomposites are elucidated from Figure 2 and Table 1. The SeNPs were effectually gathered after reduction with RA and NCt; the metal particles were uniformly distributed, negatively charged (−32 mV) and had an average size of 11.5 nm (Figure 2—Se). Regarding NCt, the polymer nanoparticles carried positive charges (+36.7 mV) and a mean diameter size of 176.2 nm. The NCt particles after encapsulation of RA/SeNPs had larger particles’ size (mean diameter = 182.6 nm) and their surface positivity was slightly lowered to become (+30.4 mV). The NCt/RA/SeNPs nanocomposite appeared with inconsistent shapes and some aggregates appeared within their matrix (Figure 2—CG).

The RA possessed high potentiality for reducing SeNPs in the current study, which could be supported by former investigations that used phytoconstituents for reducing many nanometals and validated their high efficiencies [19,20,38,39]. The increment of NCt particles’ size after conjugation with RA/SeNPs, and the reduction of their positivity, validated the effectiveness of the NCt nanopolymer to cap/encapsulate the other molecules. It was stated that the encapsulation or capping of phytocompounds and nanometals within nanopolymers derive the composited particles to have larger sizes than their parent ingredients [17,19]. Furthermore, the slight reduction in NCt/RA/SeNPs surface charge could indicate the physical capping of NCt particles to the other molecules, rather than their interaction biochemically with them [40]. The achieved zeta potentials (>+30 mV and <−30 mV) and DLS results of inspected NPs implied their prominent dispersion and stability potentialities in solutions [41]. However, the promising findings in the current investigation, especially the elevated stability and minute sizes of NCt-based nanocomposites, are supported by earlier relevant studies that employed varied chitosan types and biosynthesis promoters [17,19,40]. NCt was stated, as confirmed here, to be an ideal biopolymer for capping/stabilizing additional bioactive molecules and nanomaterials [17,40]. These important capabilities and the synergism between NCt and RA/SeNPs can warrant the good dispersion and bioactivity of the generated nanocomposite, which facilitate their actions within the liquid matrix [18,19].

### 3.3. Bioactivities of Biomaterials-Based Edible Coating

The antifungal potentialities of NCt- and NCt/RA/SeNPs-based coatings, to suppress the growth and progress of *A. alternata*, were evidenced either in vitro (determined as fungal radial growth on PDA) or on experimentally infected persimmon fruits with the phytopathogen (Table 2). The in vitro challenge revealed that NCt could effectively reduce *A. alternata* radial growth with percentages of 62.6 and 77.4%, when the PDA medium was amended with 0.5 and 1.0% from NCt, respectively. The same concentrations from NCt/RA/SeNPs in PDA led to 84.6 and 97.2% growth reduction, respectively. The standard fungicide (imazalil) amendment led to a 91.6% reduction of fungal growth, which was significantly less effective than the NCt/RA/SeNPs composite at a concentration of 1.0% (Table 2). The applications of nanocomposites-based coatings onto infected persimmon fruits could effectually protect the fruits from fungal spoilage, as evidenced by the reduction of *A. alternata* black rot incidence and severity by 91.2 and 92.4%, respectively, with the application of NCt/RA/SeNPs at 0.5% concentration. Promisingly, the NCt/RA/SeNPs nanocomposite (at 1.0% concentration) could completely prevent the black rot incidence and severity in infected fruits for the duration of 21 days; no disease signs were observed even after fruits storage for further 30 days.

Regarding the firmness of infected persimmon fruits after storage, a sharp decrease (67.4%) in fruits’ firmness was detected in water-coated (negative control) group, e.g., from 49.71 N to 16.21 N. The firmness reduction percentage was the minimum (14.3% reduction) with the treatment with NCt/RA/SeNPs coating at 1.0% concentration, whereas the firmness reductions were 30.8 and 32.7% after coating with 0.5% NCt/RA/SeNPs and 1.0% of NCt, respectively.

The antifungal potentialities of chitosan and its nanoforms (NCt) were established toward plentiful phytopathogenic species [42]. The illustrated mechanisms for these antifungal actions included: (1) the binding of positive amino groups in NCt with phospholipids on the microbial surface, which affect their functionality and permeability; (2) the penetration of NCt inside microbial cells, interaction with their DNA and RNA and prohibiting their replication/transcription; (3) the adsorption/flocculation of electronegative molecules in a microbial cell by NCt, which disturbs their physiological activities; (4) the formation of impervious polymeric layers surrounding microbial membranes by NCt, which alters cells permeability and uptake of essential nutrients; and (5) the chelating action NCt that bind metals and prohibit microbial development [20,43]. All of these factors are generated from native chitosan and strongly strengthened after its transformation to NCt [42]. Accordingly, it is suggested and validated here for NCt to perform these functions with high capability, which resulted in the effectual inhibition of *A. alternata* growth in vitro and in infected fruits.

The RA/SeNPs provided extra antifungal activity after conjugation with NCt; this is strongly attributed to their combined antimicrobial actions, which were strengthened after the composite nanoconjugation. Each of the conjugated molecules has varied modes of antimicrobial mechanisms, and the microbial cell is very difficult to resist such diverse actions [17,20].

The nanometals mechanisms of antimicrobial action (regarding different types of metals) include the NPs capability for generating ROS “reactive oxygen species”, disrupting bimolecular pathways, releasing cations, interacting/disrupting cellular membrane and depleting ATP synthesis/functions [44]. Additionally, many investigations confirmed the antifungal potentialities of diverse nanometals (including Cu, Ag, Se and TiO_2_) against phytopathogenic fungi (including *A. alternata*, *A. citri*, *P. digitatum* and *Aspergillus* spp.) [45,46,47]. They stated the concentration and size-dependent actions of nanometals to inhibit fungal growth, penetrate into their cells and disrupt their biological functions [47]. The amendment of NCt with nanometals and their employment as edible coating could be recommended to extend the shelf-life of fruits and increase their firmness; this composite has both the antifungal action of NPs and the tissue-protective action of NCt, which were reported earlier [33,48].

The RA, either alone or in conjugation with NCt, was formerly acclaimed to possess potent antifungal actions against phytopathogens [22,24]; the NCt nanoconjugation with RA could facilitate their penetration into fungal cells and disruption of their vital pathways [26]. The application of RA-NPs-based coating was significantly effective in reducing the disease incidence and severity, caused by *P. digitatum* and *A. alternata*, in tomato fruits; the effectiveness augmented with the concentration increasing of RA in coating materials [24,26]. Recently, the combination of NCt, phytocompounds and SeNPs was verified as a potent formulation to compose edible coating for phytopathogens’ control and fruits protection [20]; these compositions had the synergistic actions from each agent, including their antifungal potentialities, oxidation–reduction, tissues protection and respiration control [19,20].

### 3.4. Persimmon Fruits Quality

**Figure 3:**
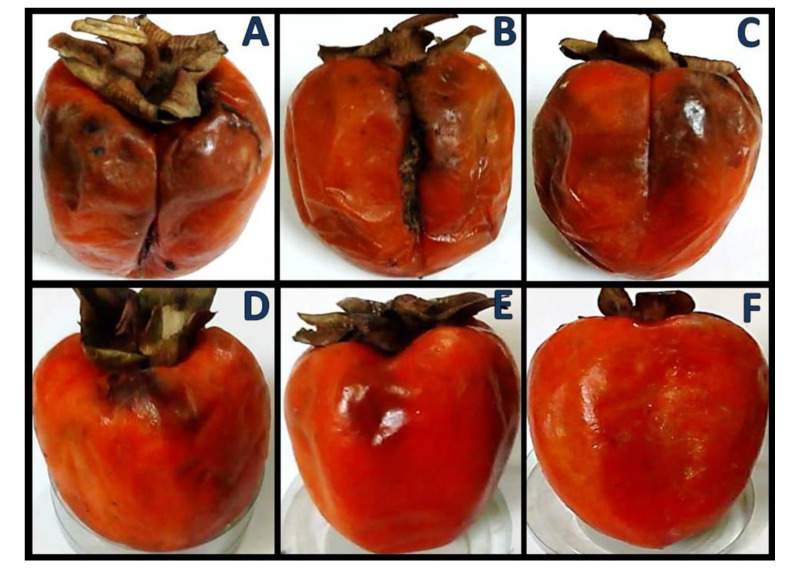
Examples of infected persimmon fruits after coating. (**A**): uncoated; (**B**): water-dipped; (**C**): water-dipped after 7 days; (**D**): coated with 1.0% nanochitosan; (**E**): coated with 0.5% nanochitosan/rosmarinic acid/Se nanocomposite; (**F**): coated with 1.0% nanochitosan/rosmarinic acid/Se nanocomposite, after 14 days of storage for treatments (**A**,**B**,**D**–**F**).

The appearance, firmness and overall quality of coated persimmon fruits were maintained throughout the storage duration for 30 days and no fungal infestation was observed, especially for coated fruits with 1% NCt/RA/SeNPs (Figure 3). The fruit coatings with nanocomposites could preserve their color and texture and prevent tissue browning, which appeared clearly in control group (Figure 3A).

The high contents of carotenoids (e.g., β-carotene, zeaxanthin, lutin and β-crytoxanthin) in persimmon fruits are accumulated throughout ripening, to transform the fruits’ color from light orange to deep reddish-orange [49]. Therefore, NCt-based coatings here could be claimed to suppress carotenoids’ accumulation, possibly by obstructing the ripening associated processes and preventing the extensive change in persimmon fruits’ color [20].

It was targeted here to maintain the firmness of persimmon fruits via their coating with formulated ECs, as the upholding of flesh firmness and stiffness is considered a key factor for augmenting quality and increasing the marketability of persimmon fruits [50]. It could be claimed from current results that ECs-treated fruits had lower respiration rates and interior enzymatic activities, which resulted in maintaining their structure and firmness. The firmness breakdown of persimmon is generally associated with intensified respiration and destroying cell wall action via enzymatic activities, which involve the fracture of tissues’ structural polysaccharides, particularly pectins and hemicelluloses [51]. The NCt-based ECs have remarkable attributes to uphold fruit firmness, as proved after their storage, compared to the untreated (control) group. Formerly, the chitosan and NCt-based ECs were stated to have an extraordinary ability for forming a thin layer on coated fruits’ surface, which can modify the interior gas exchanges (between O_2_ and CO_2_) in fruit, and directly affect fruits’ respiration rate [20]. These combined factors could lead to tissue firmness maintenance [52]. However, the current results for persimmon coating with NCt-based coating are supported by former investigations [53], which reported the firmness upholding in tomato fruits after NCt coating; they attributed the firmness maintenance to controlled gas exchanges, moisture loss and reduced respiration rate in coated fruits. Furthermore, the coating of persimmon fruits with NCt- phenylalanine nanoconjugates coating could expressively prevent the decrement of fruits firmness and reduced black rot disease incidence and severity [51]; due to the antifungal action of NCt and the coating materials’ effect on respiration rate and moisture loss. Additionally, the current findings indicated the effectiveness of NCt conjugation with RA/SeNPs for the utmost preservation of persimmon fruits. Supporting that, the NCt conjugation with metals NPs to formulate fruits coatings was reported to augment antioxidant enzymes’ actions, which resulted in maintaining the stiffness of coated fruits [54]. The formulated NCt-based ECs could be suggested to preserve the amounts of soluble tannins in treated persimmon fruits and prevent their combination with pectin within fruits’ surfaces. The soluble tannins contents in persimmon fruits (≥1000 ppm at harvesting) are the limiting factor for their firmness during storage; these tannins are combined with released pectin from cell walls and the complex is precipitated and insoluble, leading to firmness loss [55]. Furthermore, the NCt-based coatings were stated to prevent pectin from releasing from fruits tissues and hinder its interaction with tannins; thus, it can prolong fruits’ shelf lives and keep their firmness [35,51,56,57].

## 4. Conclusions

The targets of *A. alternata* black rot prevention and persimmon fruits quality maintenance were achieved via formulated edible coatings based on NCt and RA-mediated SeNPs. The nanoparticles were effectually synthesized, with minute particle sizes, and their high efficiencies were proved for inhibiting *A. alternata* growth. The most effective antifungal agent was the NCt/RA/SeNPs nanocomposite. The coating of persimmon with nanoparticles-based ECs resulted in a significant reduction of black rot disease severity and incidence in artificially infected fruits. The formulated ECs with 1% NCt/RA/SeNPs nanocomposite could completely prevent fruits from black rot incidence and severity. The firmness of fruits was greatly augmented after ECs treatments, particularly with based coatings on NCt/RA/SeNPs composite. The produced ECs in the current study, based on NCt/RA/SeNPs composite, are greatly recommended as a human-friendly matrix to prohibit *A. alternata* growth and maintain the shelf-life and quality of persimmon fruits. Further measurements of quality or other physiological parameters of ECs-treated persimmon fruits are suggested to support the research findings in future works.

## Figures and Tables

**Table 1:** The distribution of nanoparticles size and their zeta-potential for fabricated chitosan nanoparticles (NCt), selenium nanoparticles (SeNPs) and NCt/ rosmarinic acid/SeNPs nanocomposites (NCt/RA/SeNPs).

NPs	Size Range (nm)	Mean Diameter (nm)	Zeta Potential (mV)
NCt	39.6–258.5	176.2	+36.7
SeNPs	3.4–25.1	11.5	−32.2
NCt/RA/SeNPs	48.7–276.4	182.6	+30.4

**Table 2:** Impact of application with an edible coating based on nanochitosan (NCt) and its composite with rosmarinic acid/Se nanoparticles (NCt/RA/SeNPs) on the growth of *Alternaria alternata,* in vitro and on experimentally infected persimmon fruits after 21 days of storage at 15 °C.

Antifungal Compound	Conc. (%)	Reduction (%)			
		In Vitro	Coated Fruits		
		Colony Radial Growth *	Disease Incidence	Disease Severity	Firmness **
NCt	0.5	62.6	51.5	46.8	41.5
	1.0	77.4	62.9	68.4	32.7
NCt/RA/SeNPs	0.5	84.6	91.2	92.4	30.8
	1.0	97.2	100	100	14.3
Imazilil	0.5	91.6	88.6	73.4	31.2
Water ***	-	0	0	0	67.4

* The colony radial growth means were measured after incubation on PDA plates for 7 days at 25 °C. ** The mean firmness value in control samples at zero time was 49.71 N. *** Water application (negative control) exhibited colony radial growth, disease incidence and disease severity of 87.3 mm, 93.3% and 78.2%, respectively.

## Data Availability

The data presented in this study are available on request from the corresponding author.
